# Suppression of Extensive Neurofilament Phosphorylation Rescues α-Internexin/Peripherin-Overexpressing PC12 Cells from Neuronal Cell Death

**DOI:** 10.1371/journal.pone.0043883

**Published:** 2012-08-27

**Authors:** Wen-Ching Lee, Daphne Kan, Yun-Yu Chen, Shan-Kuo Han, Kuo-Shyan Lu, Chung-Liang Chien

**Affiliations:** 1 Department of Anatomy and Cell Biology, College of Medicine, National Taiwan University, Taipei, Taiwan, Republic of China; 2 Center of Genomic Medicine, College of Medicine, National Taiwan University, Taipei, Taiwan, Republic of China; Indian Institute of Toxicology Reserach, India

## Abstract

Intermediate filament (IF) overproduction induces abnormal accumulation of neuronal IF, which is a pathological indicator of some neurodegenerative disorders. In our study, α-Internexin- and peripherin-overexpressing PC12 cells (pINT-EGFP and pEGFP-peripherin) were used as models to study neuropathological pathways responsible for neurodegenerative diseases. Microarray data revealed that Cdk5-related genes were downregulated and Cdk5 regulatory subunit-associated protein 3 (GSK-3α and GSK-3β) were upregulated in pINT-EGFP cells. Increased expression of phosphorylated neurofilament and aberrant activation of Cdk5 and GSK-3β were detected in both pEGFP-peripherin and pINT-EGFP cells by Western blotting. In addition, pharmacological approaches to retaining viability of pINT-EGFP and pEGFP-peripherin cells were examined. Treatment with Cdk5 inhibitor and GSK-3β inhibitor significantly suppressed neuronal death. Dynamic changes of disaggregation of EGFP-peripherin and decrease in green fluorescence intensity were observed in pEGFP-peripherin and pINT-EGFP cells by confocal microscopy after GSK-3β inhibitor treatment. We conclude that inhibition of Cdk5 and GSK-3β suppresses neurofilament phosphorylation, slows down the accumulation of neuronal IF in the cytoplasm, and subsequently avoids damages to cell organelles. The results suggest that suppression of extensive neurofilament phosphorylation may be a potential strategy for ameliorating neuron death. The suppression of hyperphosphorylation of neuronal cytoskeletons with kinase inhibitors could be one of potential therapeutic treatments for neurodegenerative diseases.

## Introduction

Five major neuronal intermediate filament (IF) proteins have been identified in the adult mammalian central nervous system (CNS), including 66 kD α-internexin, 57 kD peripherin, and three neurofilament (NF) proteins, which are neurofilament light (NF-L, 68 kD), medium (NF-M, 145 kD), and heavy (NF-H, 200 kD) [1], [2]. Among the neuronal IFs, α-internexin is widely expressed in the adult CNS, especially in most neurons when they begin to differentiate and before the expression of the NF triplet proteins during development [3], [4], [5]. α-Internexin is recognized to be structurally and functionally associated with the NF triplet proteins in the mature CNS [6]. Peripherin is predominantly expressed in the peripheral nervous system (PNS) and in some neuronal populations of the CNS [7], [8], [9]. It has been reported that α-internexin and peripherin can self-assemble or co-assemble with neurofilament protein subunits to form the filamentous structure before their translocation into the axons and constitute a shape-maintaining IF network in mature neurons [5], [10], [11], [12], [13], [14].

Abnormal neuronal IF accumulation is a neuropathological signature of many neurodegenerative disorders, such as Alzheimer’s disease, Parkinson’s disease, dementia with Lewy bodies, and amyotrophic lateral sclerosis [5], [15], [16], [17], [18]. Overproduction of internexin and peripherin are involved in pathogenesis of neurodegenerative disorder, as their overexpression can cause a different type of neuropathy and provide additional insights into the mechanisms of neuronal dysfunction and neurodegeneration. [3], [4], [5]. α-Internexin has been identified as a major component of the pathological inclusions in frontotemporal dementia, which also called ‘neuronal intermediate filament inclusion disease (NIFID)’ [19], [20]. The signature lesion in NIFID is neuronal cytoplasmic inclusions, which contain all type IV intermediate filament proteins [19], [20], [21], [22]. Aggregates of peripherin together with other neuronal IFs were found as major components of abnormal IF inclusion bodies in mature or aging motor neurons in amyotrophic lateral sclerosis (ALS) patients [23], [24], [25]. Transgenic mice that overexpressed peripherin could develop a late-onset motor neuron death and IF inclusions resembling axonal spheroids found in ALS patients [26]. These studies indicated that abnormal neuronal IF accumulation may play a crucial role in the pathogenesis of neurodegenerative disorders.

The rat adrenal medulla pheochromocytoma PC12 cells were applied as a good cellular model for studying the pathological role of neuronal cytoskeletons in the neuronal differentiation and cell death in many studies [27], [28], [29]. Our previous work showed that overexpression of α-internexin or peripherin in PC12 cells (pINT-EGFP and pEGFP-Peri cells) enhances neurite outgrowth during the early stages of NGF induction. We also observed ultrastructurally massive IF accumulation, swelling mitochondria and degenerating neurites during the later stages of NGF?induced neuron differentiation in pINT-EGFP and pEGFP-Peri cells [29], [30].

Recently, direct evidence on the identity of phosphorylated NF proteins as an integral part of neurofibrillary tangles in AD brains was revealed by immunochemical and mass spectrometric analysis [31]. NF proteins, especially NF-M and NF-H, have many Lys-Ser-Pro (KSP) repeats in the C-terminal region that can be phosphorylated by cyclin-dependent kinase 5 (Cdk5) and glycogen synthase kinase-3 β (GSK-3β) [32], [33], [34], [35], [36], [37], [38], [39], [40], [41]. In this study, we investigated whether the inhibition of Cdk5 and GSK-3β activity would affect the hyperphosphorylation states of neuronal IF by the pharmacological approach.

To gain a better understanding of the association between neuronal cell death and excessive production of peripherin/α-internexin, we examined the neurodegeneration via overexpression of peripherin/α-internexin in PC12 cells. We aimed to discover the up-stream effectors of the IF-overexpression-induced cell death, thus microarrays were used to analyze the candidate genes triggered by overexpression of α-internexin in PC12 cells, while biochemical, cell biology, and pharmacological approaches were applied to elucidate the neuropathological mechanisms of neuronal IF accumulation.

## Materials and Methods

### Cell Culture and Drug Treatment

The rat pheochromocytoma PC12 (ATCC CRL-1721TM) and two stable clones (pEGFP-Peripherin and pINT-EGFP) established from PC12 cells were used. The latter two cells were constructed to overexpress GFP-Peripherin and internexin-GFP fusion protein respectively. Cloning of pEGFP-Peripherin and pINT-EGFP constructs were described previously [29], [30]. The adherent cells were grown in Dulbecco’s modified Eagle’s medium (DMEM) (Invitrogen, Carlsbad, CA) containing 7.5% fetal bovine serum (FBS), 7.5% horse serum (Invitrogen), and 1x antibiotic/antimycotic (Invitrogen) on the culture dishes precoated with 50 µg/ml poly-D-lyine (Sigma, St. Louis, MO). All cultures were maintained in a humidified chamber with 5% CO_2_ at 37°C. The cells were seeded at an initial density of 7.2×10^5^ cells per 60 mm dish and allowed to adhere for 2 h before neuronal differentiation induced by 100 ng/ml nerve growth factor (R&D Systems Minneapolis, MN). The medium was replaced with fresh medium containing nerve growth factor (NGF) every 2 days.

When used, the Cdk5 inhibitor roscovitine (20 µM) (Sigma-Aldrich, St. Louis, MO, USA), or the GSK-3β inhibitor SB-216763 (5 µM) (Sigma-Aldrich) was added to the medium on day 6 of NGF induction. 1% DMSO was used as the vehicle control for inhibitor treatment experiments.

### Live Cell Imaging

The pEGFP-Peripherin and pINT-EGFP cells were seeded on 35 mm μ-Dishes (Ibidi, Martinsried, Germany) at a density of 2.4×10^5^ cells per dish. Images of GFP fluorescence were recorded before drug treatment (0 hour) and after 6 h or 12 h treatment on an inverted fluorescence microscope (Leica DMR, Wetzlar, Germany) with a 20× objective.

### Image Analysis

GFP fluorescence images of the untreated and drug (inhibitor)-treated cells were taken using a TCS SP5 confocal microscope (Leica) (modified from [42]). The cells were fixed with 4% paraformaldehyde for 10 minutes on the coverslips and washed 3 times in phosphate-buffered saline (PBS). The coverslips were then mounted onto glass slides and viewed on a Leica SP5 confocal microscope under a 20× objective. The pinhole was set at 60.7 µm and the detector gain at 815 V and 30% of laser power was applied throughout each set of experiments. During image acquisition, the cells were excited with an argon ion laser emitting at 488 nm and the emission collected over a range of 505–530 nm, targeting the green fluorescence of α-internexin-EGFP. In brief, we adjusted the z axis to obtain a clear image, then defined this z axis position as 0. We then acquired a series of images with successive 0.6 µm *z* axis displacements as we scanned from the −10.0 µm to the +10.0 µm position. Fluorescent intensities were measured in 5 randomly selected fields per experiment. Quantification of green fluorescence intensity was performed using a freely available software program ImageJ (NIH, Bethesda, MD).

### Affymetrix Genechip Analysis

To compare the gene expression profiles of control PC12 cells and pINT-EGFP cells, microarray analysis was used. Total RNA was isolated with TRIzol reagent (Invitrogen) and poly A^+^ RNA purified from the total RNA using the PolyTract System from Promega (Promega, Madison, WI). Poly A^+^ RNA (2 µg) was converted to cDNA using a double-stranded cDNA Superscript Choice System (Invitrogen) and isolated by phenol-chloroform extraction using Phase Lock Gels (Eppendorf, Westbury, NY), followed by ethanol-precipitation with Pellet Paint (Novagen, Madison, WI) as carrier. The cDNA was resuspended in RNase-free water and used as a template for *in vitro* transcription in the presence of biotinylated UTP and CTP to generate labeled cRNA. The *in vitro* transcription reaction was performed using BioArray High Yield RNA Transcript Labeling Kits (Enzo Diagnostics, Farmingdale, NY). The labeled cRNA was purified using a Qiagen RNeasy Mini Kit (Qiagen, Germantown, MD), then fragmented in fragmentation buffer (40 mM Tris-acetate, pH 8.1, 10 mM KOAc, 30 mM MgOAc), and hybridized to the microarrays. Duplicate Affymetrix Rat RG_U34A Genechip arrays (Affymetrix, Santa Clara, CA) were probed with cRNA prepared from two independent mRNA samples from 4 different sources, undifferentiated PC12 cells (PC12 D0), differentiated PC12 cells on day 6 of NGF induction (PC12 D6), undifferentiated pINT-EGFP cells (pINT-EGFP D0), and differentiated pINT-EGFP on day 6 of NGF induction (pINT-EGFP D6). The microarrays were then washed and stained using a Genechip fluidics station, the DNA chips scanned at a resolution of 6 mm on an HP Gene Array scanner, and the data analyzed using Microarray Suite 4.0 Analysis software (Affymetrix).

### Antibodies

We used the mouse monoclonal antibodies anti-α-fordrin (caspase-3 substrate) and anti-peripherin (Chemicon, Temecula, CA), anti-phosphor-NF-H (SMI-36) and anti-Cdk5 (Abcam, Cambridge, UK), anti-phosphor-NF-M (RMO55) (Upstate, Lake Placid, NY), and anti-NF-H (N52), NF-M (NN18), and NF-L (NR4) (Sigma-Aldrich). The rabbit antibodies against phospho-GSK-3β (Ser9) and GSK-3β were purchased from Cell Signaling Technology (Cell Signaling, Danvers, MA, USA). Rabbit antibody against phospho-Cdk5 (Ser159) was from Santa Cruz Biotechnology (Santa Cruz, CA, USA). The mouse anti-β-actin monoclonal antibody was used as the internal control was purchased from Novus (Novus Biologicals, Littleton, CO, USA).

### Western Blot Analysis

Cells were collected and resuspended in lysis buffer as described previously [29]. After centrifugation of the lysate at 4°C and 14,000 g for 20 minutes, the supernatant was collected for soluble protein analysis, while the pellet was resuspended in SDS-urea buffer (6 M urea, 5% SDS) to extract insoluble proteins for NF protein analysis. Lysates containing 20 µg or 40 µg of protein (determined by the Bradford assay) were electrophoresed on a 8% or 15% SDS polyacrylamide gel and transferred to a polyvinylidene difluoride membrane (Millipore, Bedford, MA), which was then incubated for 1 h at room temperature with 5% skim milk in Tris-buffered saline (TBS). Proteins were detected by primary antibodies against p-NF-H, NF-H, p-NF-M, NF-M, Cdk5, p-GSK-3β (Ser9), GSK-3β (all at 1∶1000), p-Cdk5 (Ser159) (1∶500), or β-actin (1∶5000) in 5% skim milk in TBS. Horseradish peroxidase-conjugated goat anti-mouse (AP124P) or goat anti-rabbit (AP132P) were used as secondary antibodies (Chemicon) at a dilution of 1∶2000 in TBS with 5% skim milk. Western blotting luminal reagent kits (PerkinElmer, Boston, MA) and BioMax films (Kodak, Rochester, New York) were used for detection. Quantification of band intensity was performed by ImageJ.

### Immunocytochemistry

After NGF induction and with/without drug treatments, the cells were fixed in 4% paraformaldehyde for 15 minutes, washed 3 times in PBS, and covered with ice-cold methanol for 10 minutes. Primary antibodies were applied overnight at 4°C. After the cells were rinsed for 5×3 minutes with PBS, FITC-conjugated goat anti-rabbit IgG and rhodamine-conjugated goat anti-mouse IgG (Sigma-Aldrich) were applied at a 1∶200 dilution in PBS for 2 h at room temperature while the cell nucleus was labeled with fluorescent Hoechst 33342 (10 µg/mL, Sigma-Aldrich). After 5 more rinses in PBS, the cells fixed on coverslips were mounted on a slide and viewed on a TCS SP5 confocal microscope (Leica).

### Assessment of Cell Viability

The XTT assay was used to evaluate the protective effects of kinase inhibitors on pEGFP-Peripherin and pINT-EGFP cells. After various drug treatments, cell viability was evaluated by the ability of mitochondrial succinate dehydrogenase in living cells to reduce XTT (sodium 3′-1-(phenylaminocarbonyl)-3,4-tetrazolium-bis(4-methoxy-6-nitro) benzene sulfonic acid) salt (Sigma-Aldrich) to XTT formazan, measured on an ELx808 Absorbance Microplate Reader (Biotek InStruments, Winooski, VT) at 490 nm with a reference correction at 630 nm.

### Measurement of the Mitochondrial Membrane Potential (ΔΨm)

The fluorescent dye, tetramethylrhodamine methyl ester (TMRE) (Invitrogen), was added to the cells at the end of the various treatments, at a final concentration of 50 nM in HEPES buffer for 20 minutes at 37°C in a 5% CO_2_ incubator. After washing the cells, Triton X-100 (final concentration = 0.2%) was added to lyse the cells. The released TMRE from the mitochondria was measured by fluorimetric analysis using a SPECTRAmax GEMINI XS Microplate Spectrofluorometer (Molecular devices, Sunnyvale, CA) with excitation and emission wavelengths of 553 nm and 578 nm, respectively.

### Statistical Analysis

The experimental data were presented as the mean ± SEM and were evaluated for significance by un-paired *Student t-test*. Statistical significance was established at a level of *p*<0.05.

## Results

### Dysregulation of Cdk5 and GSK-3β Expression Induces NF Hyperphosphorylation in PEGFP-Peripherin and PINT-EGFP Cells

**Figure 1:**
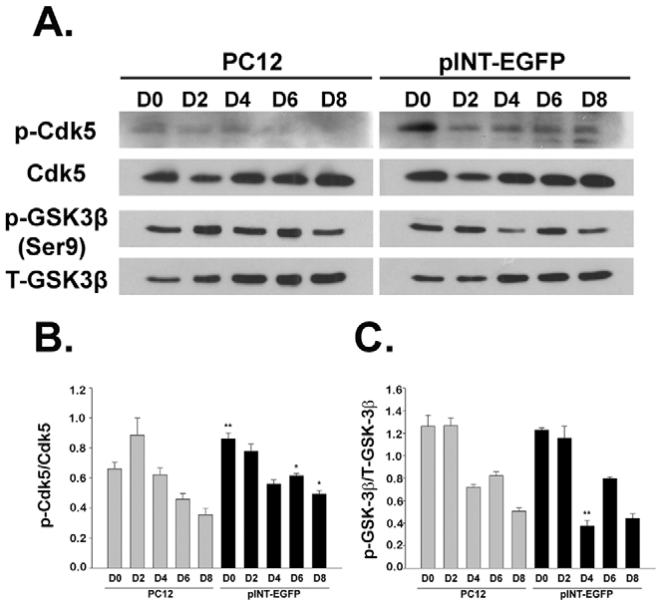
Western blot analysis of phosphorylated and non-phosphorylated Cdk5 and GSK-3β in PC12 cells and pINT-EGFP cells on days 0–8 of NGF induction. Levels of phosphorylated Cdk5 (p-Cdk5), Cdk5, phosphorylated GSK-3β (p-GSK-3β), and total GSK-3β (T-GSK-3β) analyzed by Western blotting. β-actin was used as internal control. (A) Typical Western blot. (B) Summarized results. On day 0 and day 6, p-Cdk5/Cdk5 ratios of PC12 and pINT-EGFP cells are 0.66±0.04 versus 0.86±0.04 (p<0.01), and 0.46±0.04 versus 0.62±0.02 (p<0.05), respectively (n = 3). (C) p-GSK-3β/GSK-3β ratios of PC12 and pINT-EGFP cells are 0.72±0.03 and 0.38±0.05 at day 4 of NGF induction (n = 3; p<0.01). Values are presented as the mean ± SEM for three experiments in each group. *p<0.05, **p<0.01 vs. control PC12 cells.

Previous study showed that overexpression of α-internexin and peripherin led to aberrant neuronal IF phosphorylation, neuronal IF aggregation and mislocation [29], [30] and that NF hyperphosphorylation and aberrant accumulation can be induced by inappropriate activation of several protein kinases, such as Cdk5 and GSK-3β [36], [40], [43], [44], [45]. Our microarray analysis revealed upregulation of GSK-3α and GSK-3β and downregulation of Cdk5-related genes, including Cdk5, Ab1 enzyme substrate 1, and Cdk5 regulatory subunit-associated protein 3 in pINT-EGFP cells (Table 1). These data showed that overexpression of α-internexin induced dysregulation of Cdk5 and GSK-3β. Phosphorylation of GSK-3β and Cdk5 at specific sites is able to regulate their catalytic activity. We then used Western blotting to analyze the phosphorylation of Cdk5 at Ser159 and GSK-3β at the inhibitory site Ser9 in PC12 cells, pEGFP-Peripherin and pINT-EGFP cells before and after NGF-induced neuronal differentiation [46], [47]. The intensities of the bands measured by ImageJ are listed in Table S1. As shown in Figure 1, the level of Cdk5 phosphorylation was increased in internexin-overexpressing pINT-EGFP cells. The ratio of phosphorylated Cdk5 (p-Cdk5) to non-phosphorylated Cdk5 was higher in pINT-EGFP cells than in PC12 cells at days 0 and 6 of NGF induction (Figure 1B). The phosphorylation level of GSK-3β at the inhibitory site (Ser9) in pINT-EGFP cells (p-GSK-3β/Total-GSK-3β = 0.38±0.05) was lower than that in the PC12 cells (0.72±0.03; n = 3; *p*<0.01) at day 4 of NGF induction (Figure 1A and 1C). Similar phosphorylation state of the kinases was seen in pEGFP-Peripherin cells, the phosphorylated Cdk5 (p-Cdk5) to non-phosphorylated Cdk5 ratios of PC12 cells and pEGFP-Peripherin cells at days 2, 6 and 8 of NGF induction are 1.05±0.12 versus 1.89±0.09 (*p*<0.001), 0.4±0.1 versus 0.79±0.02 (*p*<0.05) and 0.34±0.08 versus 0.6±0.03 (*p*<0.05) respectively, where n = 3 (Figure S1). In addition, p-GSK-3β to total GSK-3β ratios of PC12 cells and pEGFP-Peripherin cells are 0.97±0.04 versus 0.82±0.02 (*p*<0.05) and 0.7±0.03 versus 0.4±0.01 (*p*<0.01) at days 6 and 8 of NGF induction respectively; where n = 3 (Figure S1A and S1C). In summary, higher levels of phosphorylated Cdk5 were found in pEGFP-Peripherin and pINT-EGFP cells than those in PC12 cells. Besides, phosphorylation levels of GSK-3β at the activity inhibitory site (Ser9) were less in pEGFP-Peripherin cells and pINT-EGFP when compared to PC12 cells. The results indicated that the kinases Cdk5 and GSK-3β were more activated in the neuronal IF-overexpressing cells than in the PC12 cells.

**Table 1:** Selected differentially expressed genes in pINT-EGFP cells at day 6 of NGF induction.

Gene id.		Gene description	Regulation	Fold	*P* value
***Neuronal intermediate filament proteins***					
1371148_s_at	Internexin, alpha		Up	415.87	8.12E-06
1367845_at	Neurofilament 3, medium		Up	4.86	2.47E-07
1370058_at	Neurofilament, light polypeptide		Up	6.36	1.85E-06
1368028_at	Peripherin 1		Up	2.27	0.001266
***Calpain family of proteases***					
1387009_at	Calpain 1		Up	2.55	3.89E-04
1387860_at	Calpain 2		Up	2.31	0.00136844
***Caspase family of proteases***					
1368652_at	Caspase 9		Up	3.86	1.33E-04
1387605_at	Caspase 12		Up	7.16	9.00E-05
***Protein kinase***					
1375353_at	Cdk5 and Abl enzyme substrate 1 (predicted)		Down	3.14	0.00108826
1367879_at	Cdk5 regulatory subunit associated protein 3		Down	2.48	0.00530298
1370267_at	Glycogen synthase kinase 3 beta		Up	2.38	0.00393309
1389714_at	Glycogen synthase kinase 3 alpha		Up	2.51	0.00131834

The fold change was determined by comparing the average expression level in pINT-EGFP cells and control PC12 cells.

**Figure 2:**
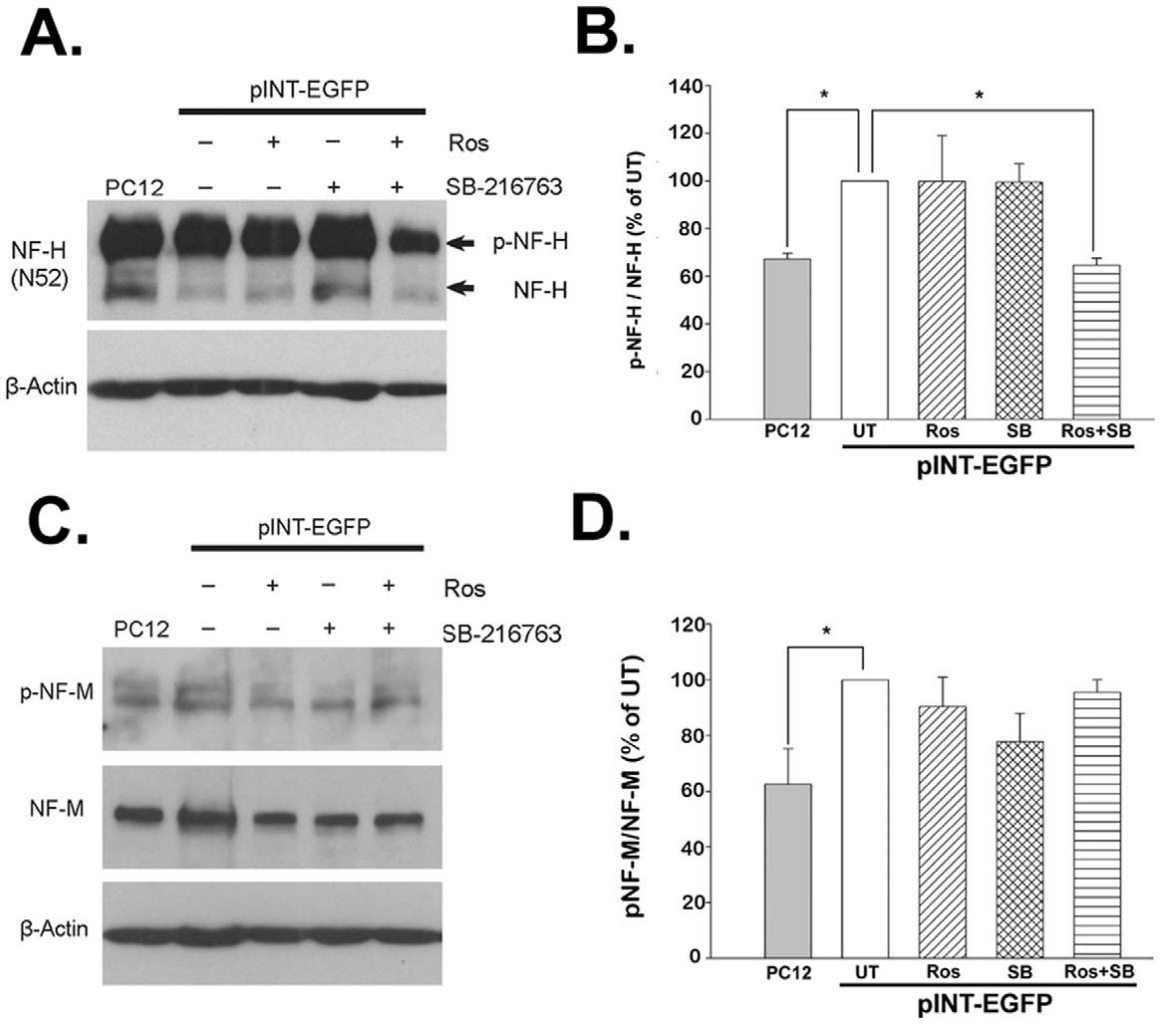
Effects of a Cdk5 inhibitor and/or a GSK-3β inhibitor on phosphorylated NF proteins in pINT-EGFP cells on day 6 of NGF induction. pINT-EGFP cells were left untreated (UT) or were treated for 24 h with 20 µM roscovitine (Ros), 5 µM SB-216763 (SB), or roscovitine plus SB-216763 (Ros+SB) on day 6 of NGF induction, then Western blotting was performed using antibody against phosphorylated NF-H (p-NF-H) or nonphosphorylated NF-H (A and B) or phosphorylated NF-M (p-NF-M) or total NF-M (C and D). The data are presented as the mean ± SEM for four independent experiments. *p<0.05 versus untreated group.

Moreover, inhibitors of Cdk5 and GSK-3β activity were used to investigate the potential influences of dysregulation of Cdk5 and/or GSK-3β on aberrant NF hyperphosphorylation in pEGFP-Peripherin and pINT-EGFP cells. After combined treatment with the Cdk5 inhibitor roscovitine (Ros) plus SB-216763 (SB), an inhibitor of GSK-3, for 24 hours started on day 6 of NGF induction, we examined the ratio of p-NF-H to nonphosphorylated NF-H (dp-NF-H) in pINT-EGFP cells. The ratio decreased to 64.64±2.86% of the phosphorylation level found in untreated cells (n = 4; *p<0.05) (Figure 2A and 2B). Combined treatment also significantly reduced phosphorylation of NF-H in pEGFP-Peripherin cells (57.83±7.66% versus untreated cells; n = 4; *p<0.05) (Figure S2A and S2B). No obvious changes of p-NF-M levels were observed in both IF overexpression cells, pINT-EGFP and pEGFP-Peripherin cells (Figure 2C, 2D and Figure S2C, 2D). Also, there were no significant changes in phosphorylation levels of neuronal IFs when individual inhibitor was applied to the pEGFP-Peripherin and pINT-EGFP cells. These data show that NF-H hyperphosphorylation is caused by dysregulation of Cdk5 and GSK-3β expression in pEGFP-Peripherin and pINT-EGFP cells.

### Effects of Roscovitine and/or SB-216763 on the Distribution of EGFP-Peripherin in PEGFP-Peripherin Cells and Internexin-EGFP in PINT-EGFP Cells

**Figure 3:**
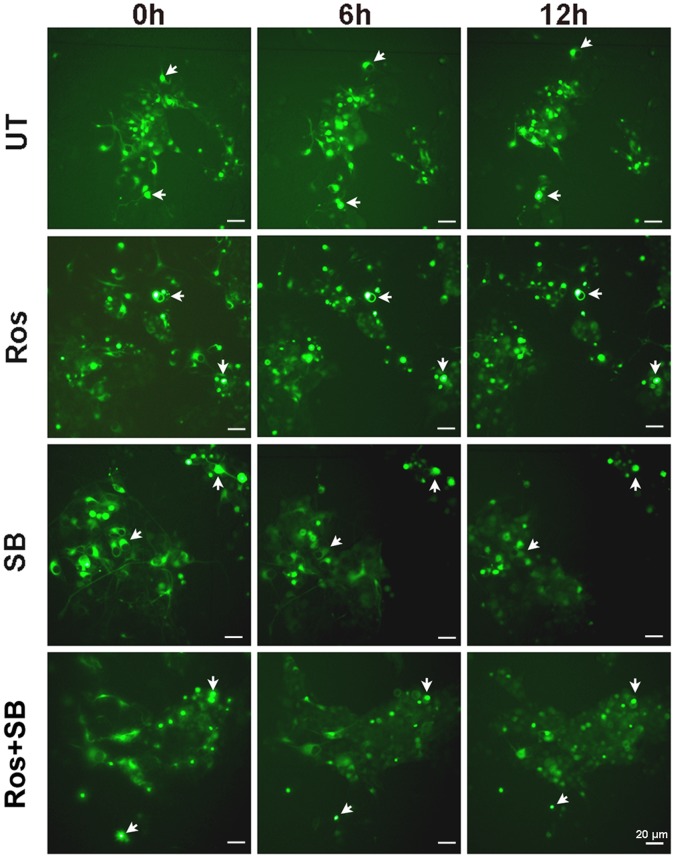
Dynamic changes in GFP fluorescence after treatment of pINT-EGFP cells with a Cdk5 inhibitor and/or a GSK-3β inhibitor. pINT-EGFP cells on day 6 of NGF induction were left untreated (UT) or were treated with 20 µM roscovitine (Ros), 5 µM SB-216763 (SB), or roscovitine plus SB-216763 (Ros+SB) as described in the legend to Figure S2 and images recorded every 6 h on an inverted fluorescence microscope. Arrows: internexin-EGFP aggregates; Original magnification: ×20, Scale bar: 20 µm.

**Figure 4:**
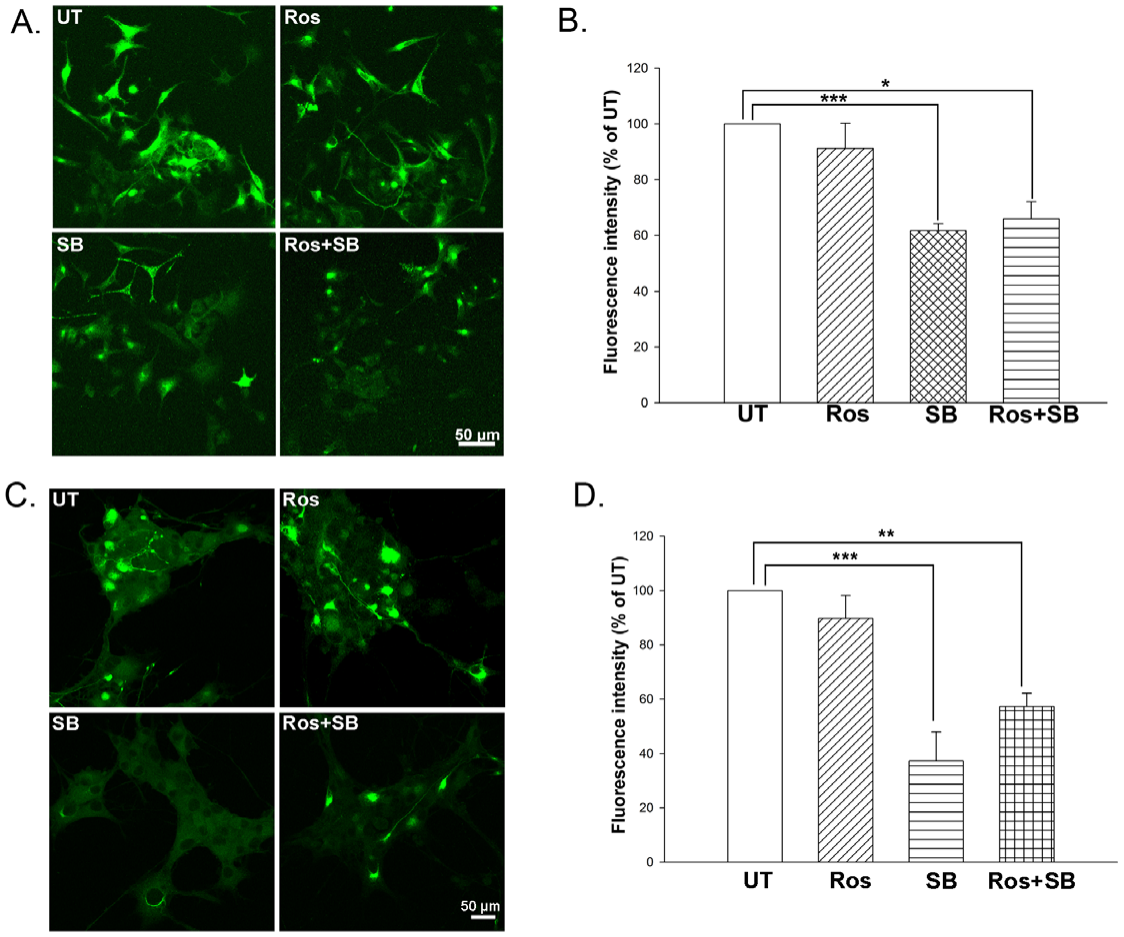
Quantification of fluorescence intensity of EGFP-Peripherin in pINT-EGFP and pEGFP-Peripherin cells treated with roscovitine (Cdk5 inhibitor) and/or SB-216763 (GSK-3β inhibitor). Untreated pINT-EGFP cells (UT) and the cells treated with roscovitine (Ros), SB-216763 (SB) or roscovitine plus SB-216763 (Ros+SB), as described in the legend to Figure 2, were examined by confocal microscopy (A) and the fluorescence intensity quantified by image analysis (B). Data for pEGFP-Peripherin cells in (C) and (D). The data are presented as the mean ± SEM for five independent experiments. ***p<0.001, **p<0.01 versus the untreated group. Original magnification: ×20, Scale bar: 50 µm.

To investigate whether inhibition of Cdk5 and GSK-3β influences neuronal IF aggregation, we treated pEGFP, pINT-EGFP and pEGFP-Peripherin cells with roscovitine (Ros) and/or SB-216763 (SB) at day 6 of NGF induction. We established pEGFP-transfected PC12 cells in our previous study [29]. We found no morphological differences between pEGFP-transfected PC12 cells and control PC12 cells. pEGFP-transfected PC12 cells (vector only, hereafter abbreviated as pEGFP cells) was used for control experiments. In NGF induction experiments, pEGFP cells also showed similar pattern with control PC12 cells. Thus we presume that overexpression of EGFP in PC12 cells has no effect on cell death or neural differentiation. Here we examined the dynamic changes of EGFP and EGPF-conjugated IF proteins (internexin-EGFP and EGFP-Peripherin) using an inverted fluorescence microscope. The morphology of PC12 cells does not change after transfected with pEGFP vector and the picture was recorded after 6-days neural induction by NGF (Figure S3). There is no aggregates of GFP protein as we examined the fluorescence pattern in the pEGFP cells (Figure S3, UT = untreated). There is no obvious changes of GFP fluorescence intensity between untreated control pEGFP cells and those after treated with SB and/or Ros (Figure S3, SB and Ros), indicating no side effect of Cdk5 and GSK-3β on the normal morphology of pEGFP-transfected PC12 cells. As seen in the fluorescence microscope images (Figure 3), SB-treated and Ros plus SB-treated pINT-EGFP cells displayed disaggregation of internexin-EGFP after 6 and 12 hours of inhibitor treatment. The same treatments on the pEGFP-Peripherin cells also caused similar disaggregation effect (Figure S4). However, no detectable change was seen in the distribution of EGFP-Peripherin and internexin-EGFP in untreated and Ros-treated cells. Furthermore, we measured fluorescence intensity of EGFP in untreated cells and inhibitor-treated cells after 24 hours inhibitor treatments. In α-internexin-overexpressing pINT-EGFP cells (Figure 4A and 4B), a significant decrease in fluorescence intensity in the SB-treated cells (62.22±9.04% versus untreated cells; n = 6; *p*<0.001) or Ros plus SB-treated cells (68.01±6.18% versus untreated cells; n = 6; *p*<0.05), while no obvious change in Ros-treated cells was found (92.61±9.04% versus untreated cells; n = 6). As shown in Figure 4C, same trend was displayed in pEGFP-Peripherin cells. Fluorescence intensity decreased in the SB-treated pEGFP-Peripherin cells (37.32±10.66% of untreated cells; n = 5; *p*<0.001) and Ros plus SB-treated pEGFP-Peripherin cells (57.33±4.8% of untreated cells; n = 5; *p*<0.01) and the change was not obvious in Ros-treated pEGFP-Peripherin cells (89.72±8.46% of untreated cells; n = 5) (Figure 4D). Our results show that inhibition of GSK-3β can promote the disaggregation of internexin-EGFP in pINT-EGFP cells and EGFP-peripherin in pEGFP-Peripherin cells. To summarize, inhibition of GSK-3β promotes the disaggregation of the intermediate filaments proteins in the cytoplasm of pEGFP-Peripherin and pINT-EGFP cells.

### Effects of the Cdk5 Inhibitor and/or GSK-3β Inhibitor on Neuron Survival and Mitochondrial Function in PEGFP-Peripherin and PINT-EGFP Cells

**Figure 5:**
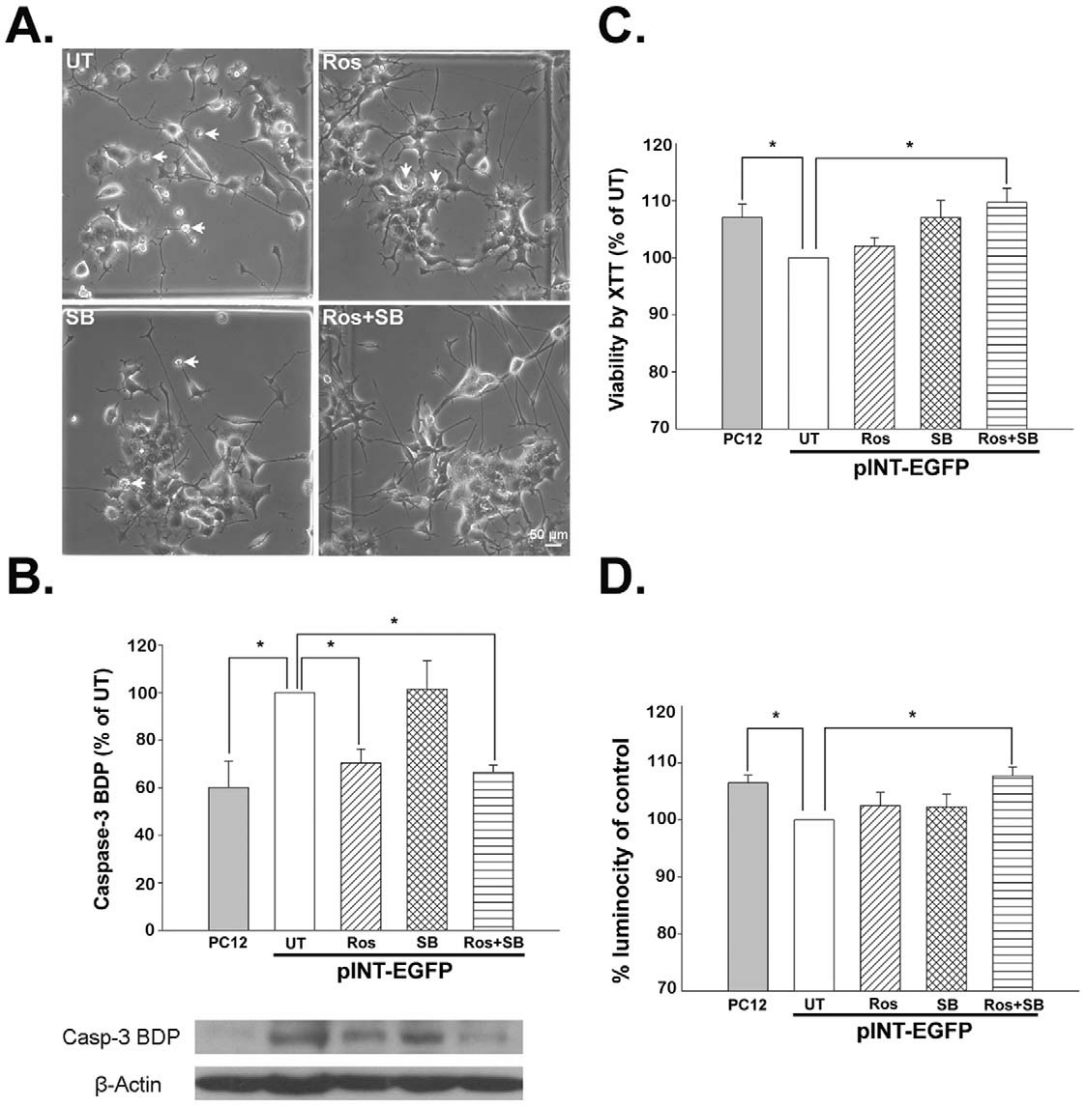
Functional effects of a Cdk5 inhibitor and/or GSK-3β inhibitor on the survival of pINT-EGFP cells. pINT-EGFP cells were left untreated (UT) or were treated with roscovitine (Ros), SB-216763 (SB) or roscovitine plus SB-216763 (Ros+SB), as described in the legend to Figure S2. (A) Cell morphology was observed under an inverted microscope. A lot of cell debris (arrows) was found in the untreated group and single inhibitor-treated cells, whereas pINT-EGFP cells treated with Ros+SB were relatively healthy. Original magnification: ×20, Scale bar: 50 µm. (B) Caspase-3 activity detected by casp-3 BDP levels. Treatment of pINT-EGFP cells with Ros plus SB also resulted in a decrease in levels of the 120 kD caspase-3 BDP (66.46±7.47% versus untreated cells; n = 6, p<0.05). A decrease in caspase-3 BDP levels was also seen with Ros treatment (70.35±14.42% versus untreated cells; n = 6, p<0.05). The data are presented as the mean ± SEM for six experiments in each group. *p<0.05 versus untreated group. (C) Cell viability evaluated by the XTT assay. A XTT assay confirmed that treatment with Ros plus SB was able to prevent cell death (109.72±2.44% versus untreated cells; n = 8, p<0.05), while treatment with Ros or SB alone had no significant effect. The data are presented as the mean ± SEM for eight experiments in each group. *p<0.05 versus the untreated group. (D) Mitochondria membrane potential evaluated by the ability of the cells to take up the fluorescent dye TMRE. treatment of pINT-EGFP cells with Ros plus SB was able to prevent the decrease in the mitochondrial membrane potential (ΔΨm) (107.68±4.39% versus untreated cells; n = 8, p<0.05). The data are presented as the mean ± SEM for eight experiments in each group. *p<0.05 versus the untreated group.

Cell morphological changes were also observed in the inhibitor-treated IF-overexpressing cells. After 24 hours Ros plus SB treatment, the pINT-EGFP (Figure 5A) and pEGFP-Peripherin cells (Figure S5A) displayed less damage when compared to the untreated cells. Caspase-3 activity was assessed by Western blot analysis of the caspase-3-specific 120 kD breakdown product (caspase-3 BDP) to examine the effect of inhibitor-treatment on cell survival. Ros plus SB treatment on pINT-EGFP cells also resulted in a decrease in the levels of caspase-3 BDP (66.46±7.47% versus untreated cells; n = 6, *p*<0.05). A decrease in caspase-3 BDP levels was also seen after Ros treatment (70.35±14.42% versus untreated cells; n = 6, *p*<0.05) (Figure 5B). As a result we found caspase-3 activity increased in IF-overexpressing induced apoptosis and the inhibitors rescued the cells from death.

An XTT assay confirmed that treatment with Ros plus SB was able to prevent cell death (109.72±2.44% versus untreated cells; n = 8, *p*<0.05), while treatment with Ros or SB alone had no significant effect (Figure 5C). In addition, treatment of pINT-EGFP cells with Ros plus SB was able to prevent the decrease in the mitochondrial membrane potential (Δ*Ψm*) (107.68±4.39% versus untreated cells; n = 8, *p*<0.05) (Figure 5D). These data show that combined treatment with Cdk5 and GSK-3β inhibitors has a rescue effect in well-differentiated pINT-EGFP cells.

After the combined treatment (Ros plus SB), we found the caspase-3 BDP decreased to 68.98±8.14% of untreated cells (n = 4, *p*<0.01). The caspase-3 BDP levels also dropped to 83.17±9.43% of untreated cells (n = 4, *p*<0.05) when treated with Ros (Figure S5B). XTT assays confirmed that treatments with Ros and Ros plus SB was able to prevent cell death as the cell viability increased to 126.92±4.34% (*p*<0.001, n = 8) and 118.21±5.01% of untreated cells (*p*<0.05, n = 8). No significant effect was observed after treatment with Ros or SB alone (Figure S6C). Cells might encounter loss of mitochondrial membrane potential (MMP, Δ*Ψm*) during apoptosis induced by neuronal IF hyperphosphorylation in pEGFP-Peripherin and pINT-EGFP cells. We then measured MMP as an important parameter of mitochondrial function and used it as an indicator of cell health. As expected, pEGFP-Peripherin cells had lower MMP than PC12 cells. Treatment of pEGFP-Peripherin cells with Ros plus SB was able to prevent the deduction in the mitochondrial membrane potential (Δ*Ψm* = 106.46±0.97% of untreated cells; n = 8, *p*<0.01) (Figure S6D). Cell death was also reduced in the pEGFP-Peripherin cells by treating with the inhibitors described above; see Figure S6. These data show that combined treatment with Cdk5 and GSK-3β inhibitors has a rescue effect in well-differentiated pEGFP-Peripherin and pINT-EGFP cells.

## Discussion

In our earlier work, the pEGFP-Peripherin and pINT-EGFP cell models were used to study neuropathological pathways responsible for neurodegenerative diseases. We reported that overexpression of either peripherin or α-internexin enhances neurite outgrowth during the early stages of NGF induction, and that massive IF accumulation, swelling mitochondria, and degenerating neurites are observed ultrastructurally during the later stages of NGF induction in the IF-overexpressing PC12 cells [29], [30]. Increased neurofilament hyperphosphorylation and abnormal accumulation of neuronal IF were also found in these cells (Figure 4A untreated cells), and this is a conspicuous feature in many human neurodegenerative diseases [5], [15], [16], [17], [18]. It has been reported that cultured motor neurons microinjected with an expression vector coding for the peripherin gene evoked an apoptotic cell death [48]. A study indicated that overexpressing peripherin can cause defective axonal transport of type IV neurofilament proteins in cultured DRG neurons from peripherin transgenic embryos [49]. Our two independent PC12 cells overexpressed of either peripherin or α-internexin also show activation of caspase-3 and possibly other related genes (such as calpain, caspase-9 and caspase-12) which trigger apoptosis and lead to neuronal death. In our previous study, we found that active calpain, caspase 12, caspase 9 and caspase-3 increased in EGFP-Peripherin-overexpressing cells and led to induction of apoptosis. After applying calpeptin (an inhibitor of calpain), caspase-3 activation in pEGFP-Peripherin cells was blocked by and neuronal cell death was suppressed in well-differentiated pEGFP-Peripherin cells [30].

**Figure 6:**
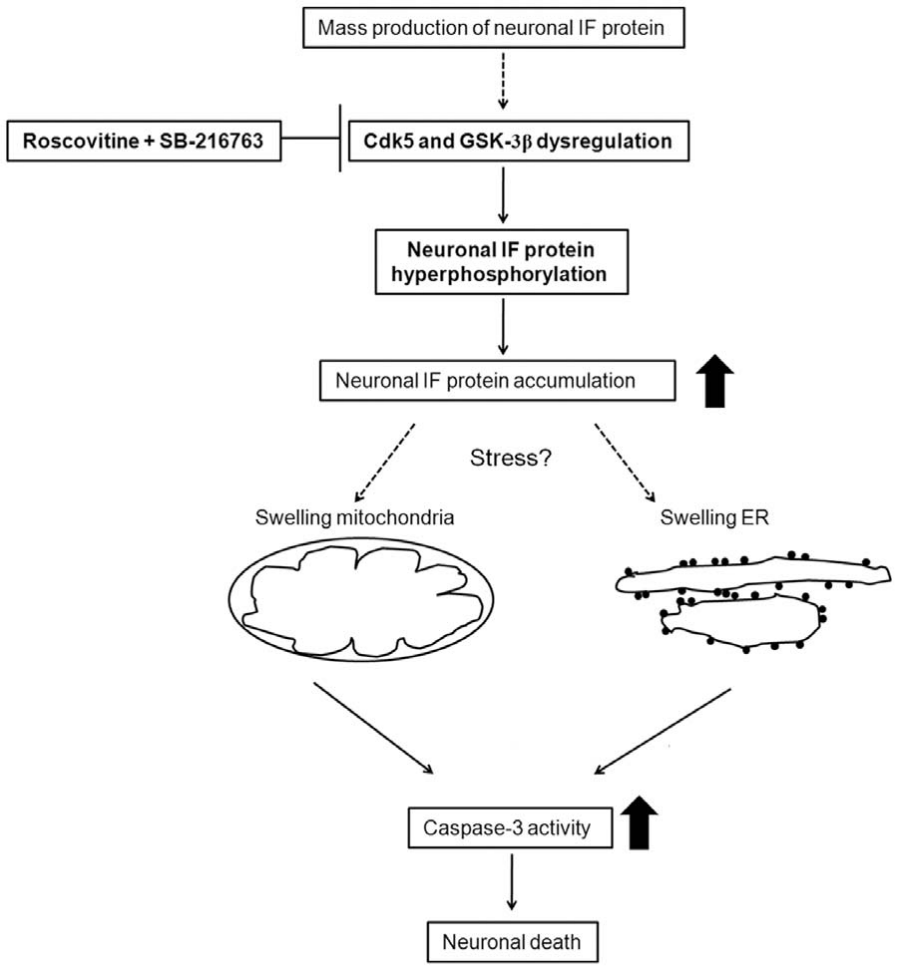
Hypothetical scheme for the accumulation of neuronal IF proteins and the subsequent induced cellular apoptosis in the pINT-EGFP and pEGFP-Peri cell models.

We propose a hypothetical scheme for neuronal death in our pEGFP-Peripherin and pINT-EGFP cell models to conclude the present data and our previous work [29], [30] (Figure 6). Overexpression of peripherin/α-internexin causes abundant neuronal IF accumulation in the cytoplasm, which deregulates Cdk5 and GSK-3β, and this triggers hyperphosphorylation of NF proteins. Phosphorylation defines the nature of NF interactions with one another and with other cytoskeletal components [50]. When NF proteins are hyperphosphorylated, they can not be transported to the axon and form aggregates. Subsequently, mitochondria and ER are trapped in the neuronal IF aggregates and indirectly damaged. We suggest that activation of calpain, caspase-12, caspase-9, and caspase-3 are involved in the dysfunction of the ER and mitochondria in our pEGFP-Peripherin and pINT-EGFP cells cell models.

The factors involved in the NF hyperphosphorylation were identified in this study. Microarray data showed that Cdk5 gene was down-regulated and GSK-3β gene was up-regulated in the pINT-EGFP cells. Moreover, the ratios of activated Cdk5 and GSK-3β were higher in peripherin/α-internexin-overexpressing cells than PC12 cells (Figure 1). These could be the reasons to hyperphosphorylation of NFs, abnormal NF accumulation and later neuronal death seen in well-differentiated pEGFP-Peripherin and pINT-EGFP cells. Thus, we examined if Cdk5 and GSK-3β participated in abnormal neuronal IF aggregation and the later neuronal death in well-differentiated pEGFP-Peripherin and pINT-EGFP cells. In order to find a solution to rescue the cells from neuronal death by dysregulating upstream effectors, we examined pharmacological approaches to preventing neuronal death using the inhibitors of the kinases Cdk5 and GSK-3β. When both of the kinases Cdk5 and GSK-3β were inhibited by their specific inhibitors Ros and SB, the aberrant NF-H hyperphosphorylation were reduced (Figure 2) and the abnormal accumulated intermediate filaments were disaggregated (Figure 3). Inhibition of GSK-3β alone was also able to promote the disaggregation of EGFP-peripherin and internexin-EGFP in pEGFP-Peripherin cells and pINT-EGFP cells (Figures 3 and 4). These results imply that preventing extensive NF phosphorylation could lead to disaggregation of neuronal IF. Furthermore, combined treatments with Cdk5 and GSK-3β inhibitors resulted in a significant decrease in p-NF-H levels (Figure 2) and a rescue effect (Figure 5) in pINT-EGFP cells as in pEGFP-Peripherin cells. Here we propose the inhibition of the kinases Cdk5 and GSK-3β which is a better approach to cell rescue in comparison with the inhibition of downstream calpain and caspases. It is likely that the accumulation of p-NF-H in the perikarya plays a crucial role in the pathogenesis of neurodegeneration. This idea is also supported by the fact that deletion of the C-terminal region of NF-H delays motor neuron pathology in a murine model of amyotrophic lateral sclerosis [51].

Transgenic mice overexpressing wild-type mouse NF-H or NF-M show neither muscle atrophy nor motor neuron loss, despite prominent axonal swelling and perikaryal neurofilament accumulation in motor neurons [52], [53]. However, overexpression of peripherin developed a late-onset motor neuron death and IF inclusions resembling axonal spheroids found in ALS patients [26]. Since neuronal IF accumulation is seen in the perikarya and axons of affected motor neurons in SOD1 mutant transgenic mice [54], [55], this transgenic mouse model will be a good candidate for confirming the therapeutic effects of protein kinase inhibitors *in vivo*. Additionally, both the ubiquitin-proteasome system and the autophagy-lysosomal system are important in protein degradation in neuronal metabolism [56]. Our pINT-EGFP and pEGFP-Peripherin cell models could provide a good alternative system to the SOD1 mutant mice *in vivo* model and can be used to study the protein degradation machinery and elucidate the complex neuropathological underlying mechanisms of neuronal cell death.

## Supporting Information

Figure S1 **Western blot analysis of phosphorylated and non-phosphorylated Cdk5 and GSK-3β in PC12 cells and pEGFP-Peripherin cells on days 0 to 8 of NGF induction.** Levels of phosphorylated Cdk5 (p-Cdk5), Cdk5, phosphorylated GSK-3β (p-GSK-3β), and total GSK-3β (T-GSK-3β) analyzed by Western blotting. (A) Typical Western blot. (B) and (C) Summarized results. Values are presented as the mean ± SEM for three experiments in each group. *p<0.05, **p<0.01, ***p<0.001 vs. control PC12 cells.
(TIF)

Figure S2 **Effects of roscovitine (a Cdk5 inhibitor) and/or SB-216763 (a GSK-3β inhibitor) on phosphorylated NF proteins in pEGFP-Peripherin cells on day 6 of NGF induction.** pEGFP-Peripherin cells were left untreated (UT) or were treated for 24 h with 20 µM roscovitine (Ros), 5 µM SB-216763 (SB), or roscovitine plus SB-216763 (Ros+SB) on day 6 of NGF induction. (A) & (B) Western blotting was performed using N52 antibody against phosphorylated NF-H (p-NFH) and nonphosphorylated NF-H (dp-NFH). (C) & (D) Phosphorylated NF-M (p-NF-M) and total NF-M recognized by antibodies RMO55 and NN18. The data are presented as the mean ± SEM for four independent experiments. *p<0.05 versus untreated group.
(TIF)

Figure S3 **GFP fluorescence after treatment of pEGFP-transfected PC12 cells (pEGFP cells) with Ros (a Cdk5 inhibitor) and/or SB (a GSK-3β inhibitor).** The morphology of untreated pEGFP cells is similar to PC12 cells. No significant changes in GFP fluorescence in pEGFP cells after inhibitor treatments. UT = untreated.
(TIF)

Figure S4 **Dynamic changes in GFP fluorescence after treatment of pEGFP-peripherin cells with roscovitine (Cdk5 inhibitor) and/or SB-216763 (GSK-3β inhibitor).** pEGFP-peripherin cells on day 6 of NGF induction were left untreated (UT) or were treated with 20 µM roscovitine (Ros), 5 µM SB-216763 (SB), or roscovitine plus SB-216763 (Ros-SB) as described in the legend to Fig. 2 and images recorded every 6 h on an inverted fluorescence microscope. Scale bar = 20 µm.
(TIF)

Figure S5 **Functional effects of a Cdk5 inhibitor and/or GSK-3β inhibitor on the survival of pEGFP-Peripherin cells.** pEGFP-Peripherin cells were left untreated (UT) or were treated with roscovitine (Ros), SB-216763 (SB) or roscovitine plus SB-216763 (Ros+SB), as described in the legend to Fig. 2. (A) Cell morphology was observed under an inverted microscope. A lot of cell debris was found in the untreated group and single inhibitor-treated cells, whereas pEGFP-Peripherin cells treated with Ros+SB were relatively healthy. Scale bar = 50 µm. (B) Caspase-3 activity detected by caspase-3 BDP levels. The data are presented as the mean ± SEM for six experiments in each group. *p<0.05, **p<0.01 versus untreated group. (C) Cell viability evaluated by the XTT assay. The data are presented as the mean ± SEM for 8 experiments in each group. ***p<0.001, *p<0.05 versus the untreated group. (D) Mitochondria membrane potential (MMP) evaluated by the ability of the cells to take up the fluorescent dye TMRE. The data are presented as the mean ± SEM for eight experiments in each group. **p<0.01 versus the untreated group.
(TIF)

Figure S6 **Western blot analysis of neurofilament proteins and CDK5.** Neurofilament proteins with and without phosphorylation from undifferentiated and NGF-differentiated PC12 cells and pINT-EGFP cells were analyzed. Overexpressed α-internexin induced neurofilament hyperphosphorylation in pINT-EGFP transfected PC12 cells. The protein level of CDK5 is similar between control PC12 and pINT-EGFP transfected cells.
(TIF)

Table S1 **Band intensities on the Western blot of phosphorylated and non-phosphorylated Cdk5 and GSK-3β in PC12 cells and pINT-EGFP cells on days 0–8 of NGF induction.** Data was obtained using ImageJ.
(DOCX)
