# Supplementary figures and images for: Suppression of Extensive Neurofilament Phosphorylation Rescues α-Internexin/Peripherin-Overexpressing PC12 Cells from Neuronal Cell Death
Source: PLoS One. 2012 Aug 27;7(8):e43883. doi: 10.1371/journal.pone.0043883 (PMC3428284; doi:10.1371/journal.pone.0043883)

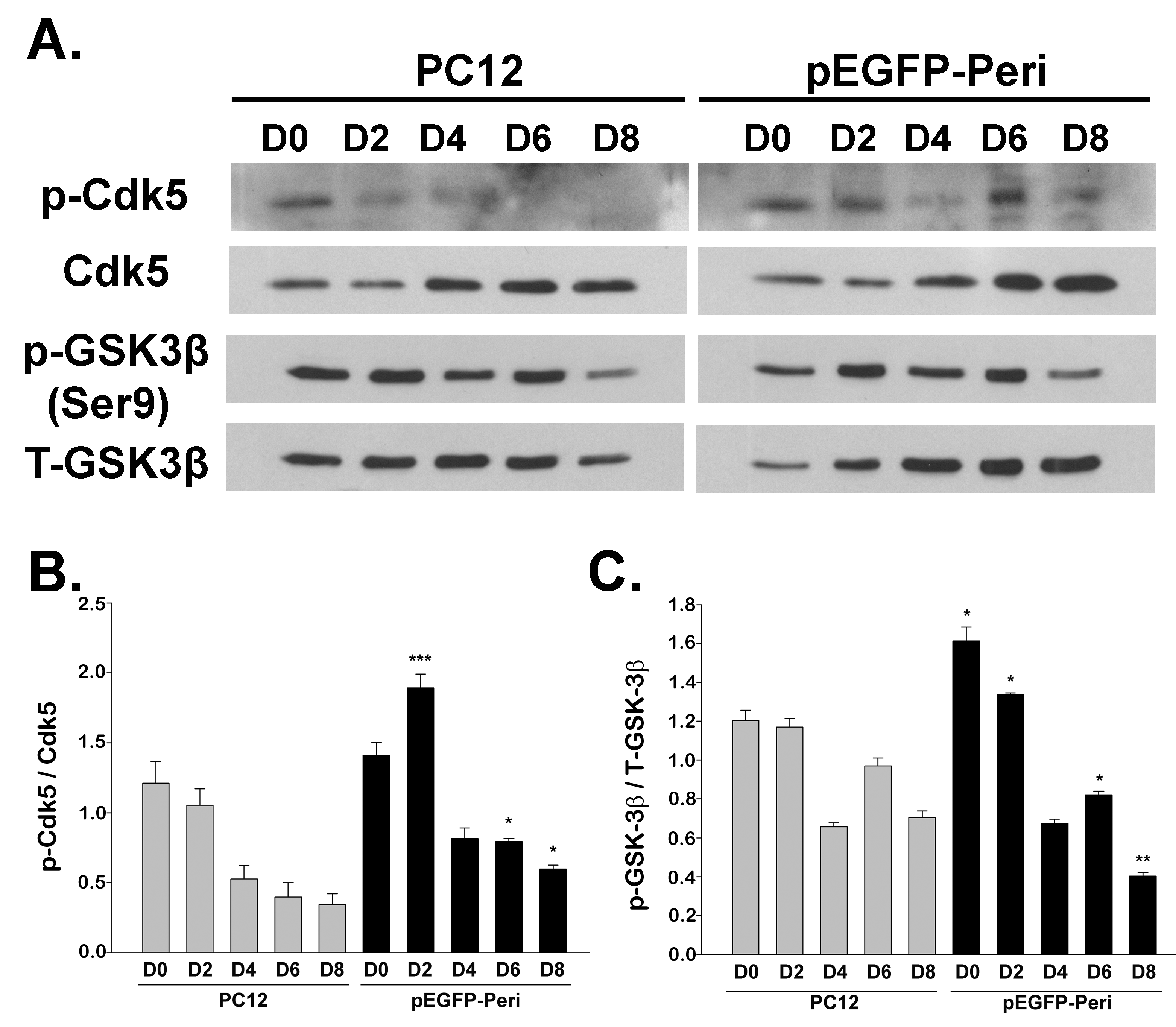

Supplement: Figure S1 — Western blot analysis of phosphorylated and non-phosphorylated Cdk5 and GSK-3β in PC12 cells and pEGFP-Peripherin cells on days 0 to 8 of NGF induction. Levels of phosphorylated Cdk5 (p-Cdk5), Cdk5, phosphorylated GSK-3β (p-GSK-3β), and total GSK-3β (T-GSK-3β) analyzed by Western blotting. (A) Typical Western blot. (B) and (C) Summarized results. Values are presented as the mean ± SEM for three experiments in each group. *p<0.05, **p<0.01, ***p<0.001 vs. control PC12 cells. (TIF) [file pone.0043883.s001.tif]

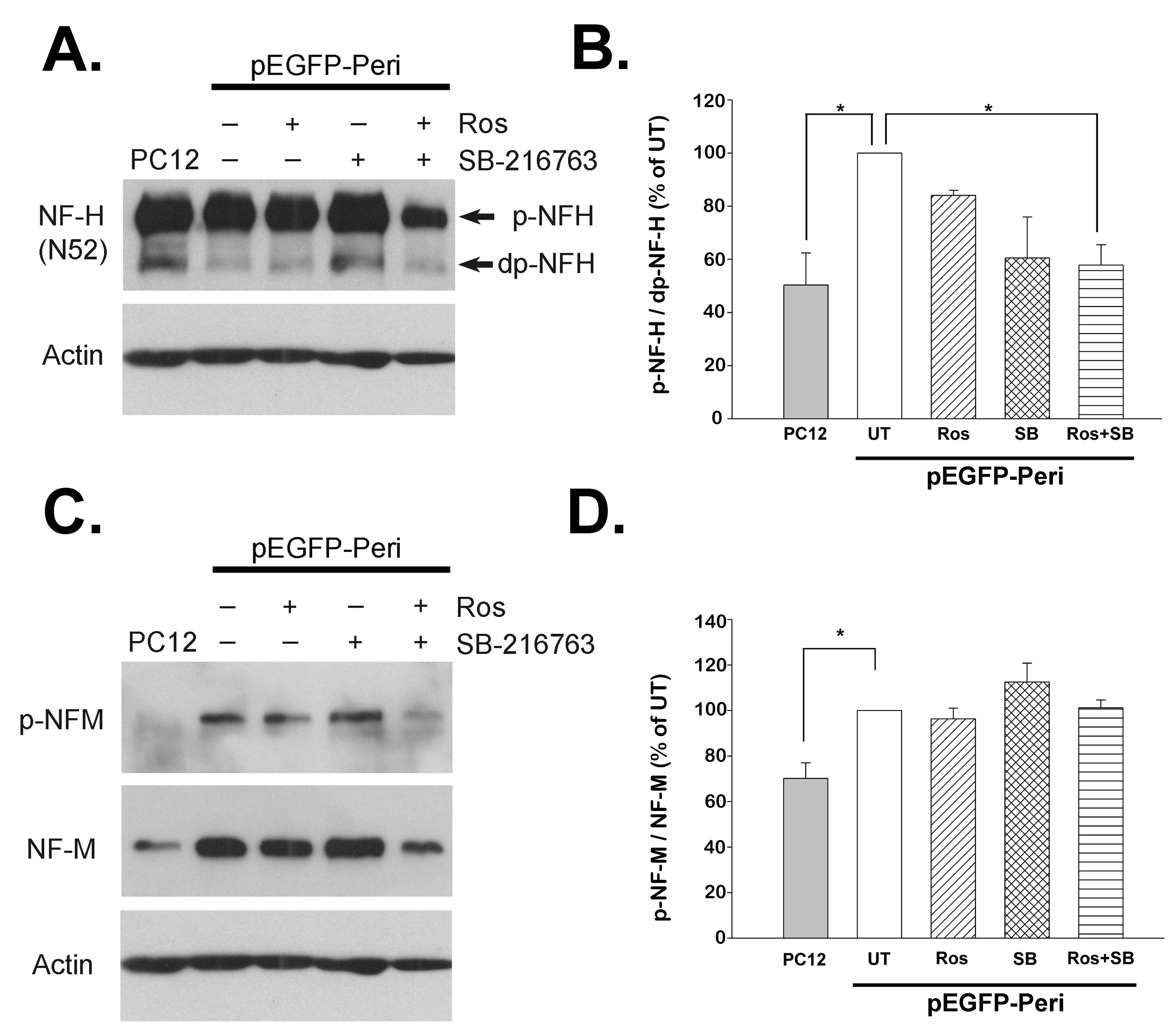

Supplement: Figure S2 — Effects of roscovitine (a Cdk5 inhibitor) and/or SB-216763 (a GSK-3β inhibitor) on phosphorylated NF proteins in pEGFP-Peripherin cells on day 6 of NGF induction. pEGFP-Peripherin cells were left untreated (UT) or were treated for 24 h with 20 µM roscovitine (Ros), 5 µM SB-216763 (SB), or roscovitine plus SB-216763 (Ros+SB) on day 6 of NGF induction. (A) & (B) Western blotting was performed using N52 antibody against phosphorylated NF-H (p-NFH) and nonphosphorylated NF-H (dp-NFH). (C) & (D) Phosphorylated NF-M (p-NF-M) and total NF-M recognized by antibodies RMO55 and NN18. The data are presented as the mean ± SEM for four independent experiments. *p<0.05 versus untreated group. (TIF) [file pone.0043883.s002.tif]

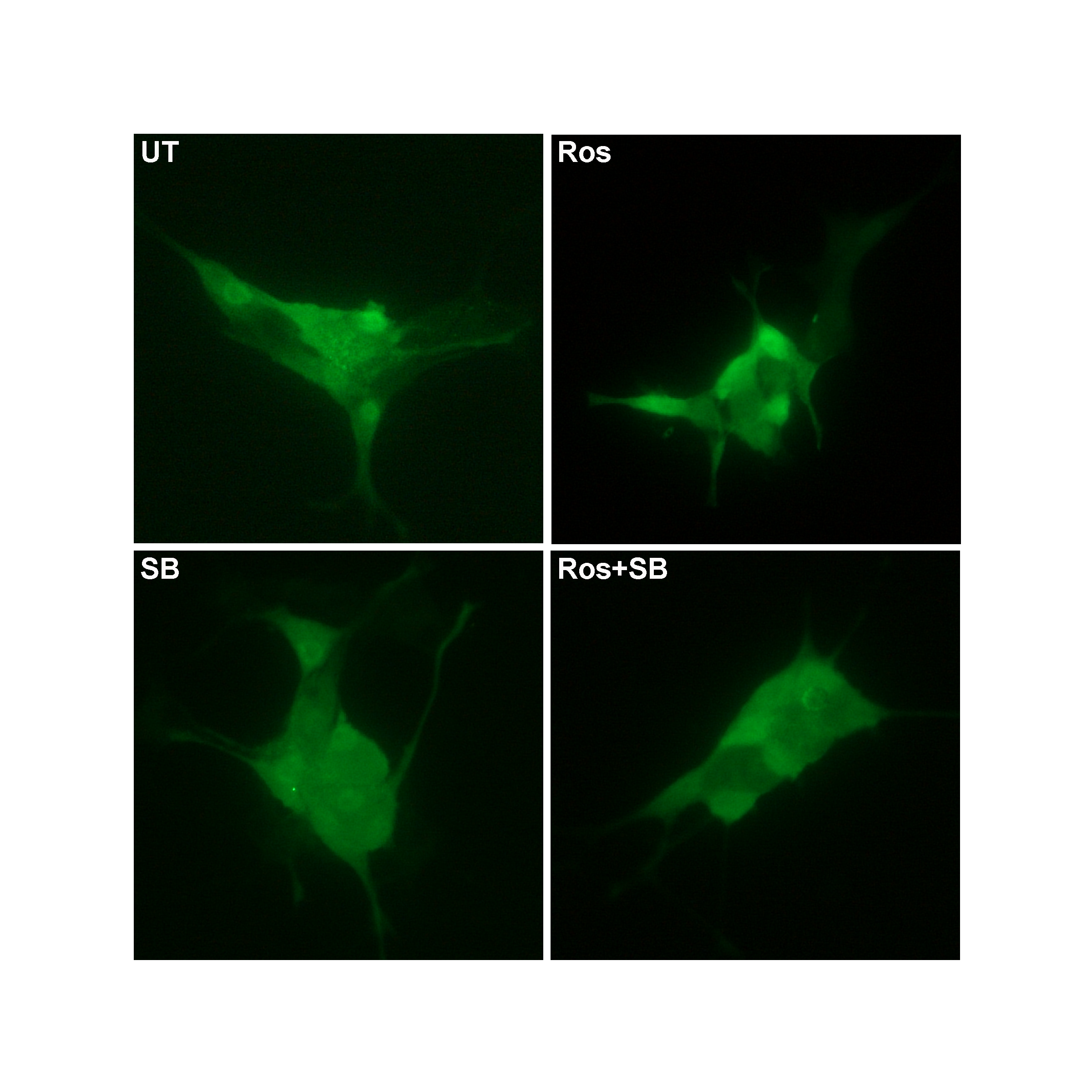

Supplement: Figure S3 — GFP fluorescence after treatment of pEGFP-transfected PC12 cells (pEGFP cells) with Ros (a Cdk5 inhibitor) and/or SB (a GSK-3β inhibitor). The morphology of untreated pEGFP cells is similar to PC12 cells. No significant changes in GFP fluorescence in pEGFP cells after inhibitor treatments. UT = untreated. (TIF) [file pone.0043883.s003.tif]

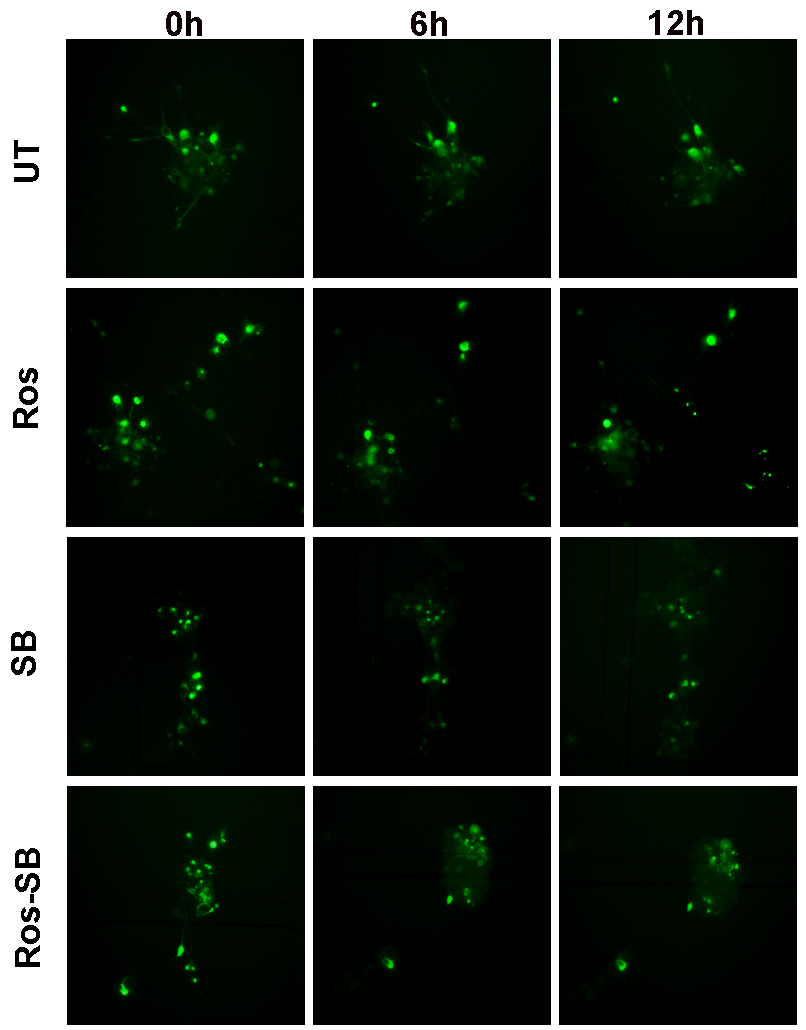

Supplement: Figure S4 — Dynamic changes in GFP fluorescence after treatment of pEGFP-peripherin cells with roscovitine (Cdk5 inhibitor) and/or SB-216763 (GSK-3β inhibitor). pEGFP-peripherin cells on day 6 of NGF induction were left untreated (UT) or were treated with 20 µM roscovitine (Ros), 5 µM SB-216763 (SB), or roscovitine plus SB-216763 (Ros-SB) as described in the legend to Fig. 2 and images recorded every 6 h on an inverted fluorescence microscope. Scale bar = 20 µm. (TIF) [file pone.0043883.s004.tif]

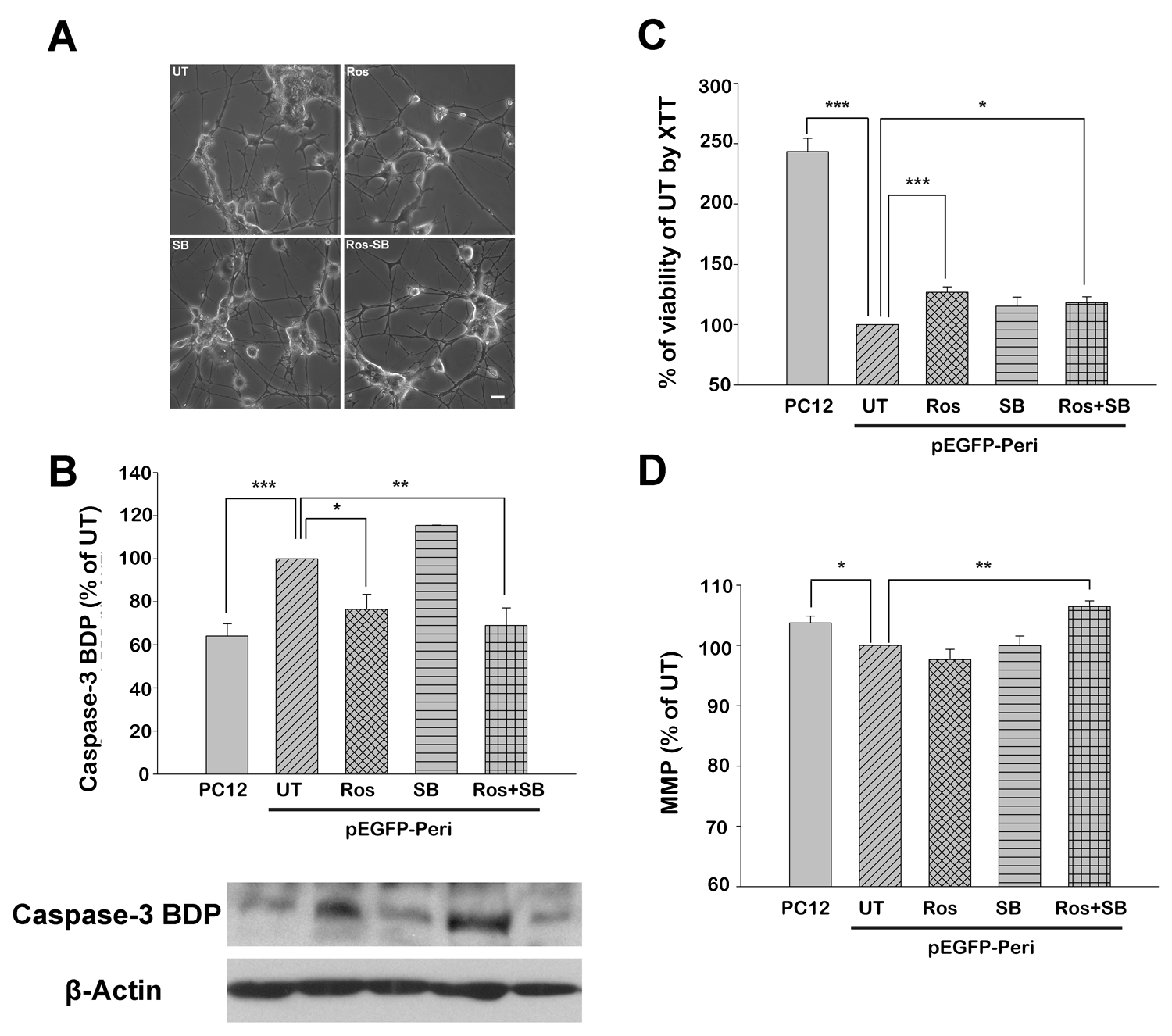

Supplement: Figure S5 — Functional effects of a Cdk5 inhibitor and/or GSK-3β inhibitor on the survival of pEGFP-Peripherin cells. pEGFP-Peripherin cells were left untreated (UT) or were treated with roscovitine (Ros), SB-216763 (SB) or roscovitine plus SB-216763 (Ros+SB), as described in the legend to Fig. 2. (A) Cell morphology was observed under an inverted microscope. A lot of cell debris was found in the untreated group and single inhibitor-treated cells, whereas pEGFP-Peripherin cells treated with Ros+SB were relatively healthy. Scale bar = 50 µm. (B) Caspase-3 activity detected by caspase-3 BDP levels. The data are presented as the mean ± SEM for six experiments in each group. *p<0.05, **p<0.01 versus untreated group. (C) Cell viability evaluated by the XTT assay. The data are presented as the mean ± SEM for 8 experiments in each group. ***p<0.001, *p<0.05 versus the untreated group. (D) Mitochondria membrane potential (MMP) evaluated by the ability of the cells to take up the fluorescent dye TMRE. The data are presented as the mean ± SEM for eight experiments in each group. **p<0.01 versus the untreated group. (TIF) [file pone.0043883.s005.tif]

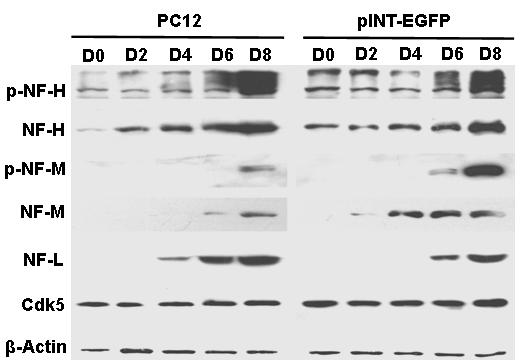

Supplement: Figure S6 — Western blot analysis of neurofilament proteins and CDK5. Neurofilament proteins with and without phosphorylation from undifferentiated and NGF-differentiated PC12 cells and pINT-EGFP cells were analyzed. Overexpressed α-internexin induced neurofilament hyperphosphorylation in pINT-EGFP transfected PC12 cells. The protein level of CDK5 is similar between control PC12 and pINT-EGFP transfected cells. (TIF) [file pone.0043883.s006.tif]
