# Supplementary material for: Suppression of Extensive Neurofilament Phosphorylation Rescues α-Internexin/Peripherin-Overexpressing PC12 Cells from Neuronal Cell Death
Source: PLoS One. 2012 Aug 27;7(8):e43883. doi: 10.1371/journal.pone.0043883 (PMC3428284; doi:10.1371/journal.pone.0043883)
Supplement: Table S1 — Band intensities on the Western blot of phosphorylated and non-phosphorylated Cdk5 and GSK-3β in PC12 cells and pINT-EGFP cells on days 0–8 of NGF induction. Data was obtained using ImageJ. (DOCX) [file pone.0043883.s007.docx]

**Suppression of extensive neurofilament phosphorylation rescues α-internexin/peripherin-overexpressing PC12 cells from neuronal cell death**

**Supporting information**

**p-cdk5/cdk5**

|  | PC12 d0 | PC12 d2 | PC12 d4 | PC12 d6 | PC12 d8 | INT d0 | INT d2 | INT d4 | INT d6 | INT d8 |
| --- | --- | --- | --- | --- | --- | --- | --- | --- | --- | --- |
|  | 0.7072 | 0.9087 | 0.6256 | 0.4576 | 0.3818 | 0.8542 | 0.7844 | 0.5935 | 0.635 | 0.5121 |
|  | 0.5714 | 0.6792 | 0.5415 | 0.3939 | 0.2711 | 0.7954 | 0.6882 | 0.5071 | 0.5849 | 0.45 |
|  | 0.7044 | 1.0711 | 0.6985 | 0.5251 | 0.4135 | 0.9331 | 0.8591 | 0.5823 | 0.6251 | 0.52 |
| Mean | 0.661 | 0.8863 | 0.6219 | 0.4589 | 0.3555 | 0.8609 | 0.7772 | 0.561 | 0.615 | 0.494 |
| SEM | 0.0448 | 0.1137 | 0.0454 | 0.0379 | 0.0432 | 0.0399 | 0.0495 | 0.0271 | 0.0153 | 0.0221 |

**p-GSK-3/GSK-3**

|  | PC12 d0 | PC12 d2 | PC12 d4 | PC12 d6 | PC12 d8 | INT d0 | INT d2 | INT d4 | INT d6 | INT d8 |
| --- | --- | --- | --- | --- | --- | --- | --- | --- | --- | --- |
|  | 1.4508 | 1.2645 | 0.7323 | 0.8843 | 0.5267 | 1.2142 | 1.1566 | 0.3436 | 0.7854 | 0.4407 |
|  | 1.125 | 1.383 | 0.7549 | 0.7651 | 0.5457 | 1.2082 | 1.3382 | 0.4727 | 0.8236 | 0.5154 |
|  | 1.2074 | 1.1546 | 0.6698 | 0.8142 | 0.4467 | 1.2627 | 0.978 | 0.315 | 0.781 | 0.3767 |
| Mean | 1.2611 | 1.2674 | 0.719 | 0.8212 | 0.5064 | 1.2284 | 1.1576 | 0.3771 | 0.7967 | 0.4443 |
| SEM | 0.0978 | 0.0659 | 0.0255 | 0.0346 | 0.0303 | 0.0172 | 0.104 | 0.0485 | 0.0135 | 0.0401 |

**Table S1. Band intensities on the Western blot of phosphorylated and non-phosphorylated Cdk5 and GSK-3β in PC12 cells and pINT-EGFP cells on days 0-8 of NGF induction. Data was obtained using ImageJ.**
